# Supplementary material for: Metabolic Transition of Milk Triacylglycerol Synthesis in Response to Varying Levels of Three 18-Carbon Fatty Acids in Porcine Mammary Epithelial Cells
Source: Int J Mol Sci. 2021 Jan 28;22(3):1294. doi: 10.3390/ijms22031294 (PMC7866201; doi:10.3390/ijms22031294)
Supplement: Supplementary file 1 [file ijms-22-01294-s001.pdf]

Supplemental Table S1. Characteristics of primers used for real-time quantitative PCR analysis

Gene	NCBI GenBank	Primer Sequence (5′→3′)	Amplicon (bp) [1]
CD36	DQ192230.1	GGACTCATTGCTGGTGCTGT<br>GTCTGTAAACTTCCGTGCCTGT	169
ACSL3	NM_001143698.1	ACCCTGGATGTGATACGCTA<br>AGTTCCCAAGAATAACCTTTT	150
FABP3	AY569332.1	CTGGGAGTGGAGTTTGATGAGAC<br>CCATGGGTGAGTGTCAGGAT	164
ACACA	NM_001114269.1	ACATCCCCACGCTAAACA<br>AGCCCATCACTTCATCAAAG	186
FASN	NM_001099930.1	GCTTGTCCTGGGAAGAGTGTA<br>AGGAACTCGGACATAGCGG	115
SCD	NM_213781.1	TGACCTAAAAGCCGAGAA<br>GCACGATGGCGTAACGAAGA	164
GPAM	XM_001927875.1	ACTATCTCCTGCTCACTTTCA<br>CGTCTCATCTAGCCTCCGTC	146
AGPAT1	EU282358.1	CCTTCTACAACGGCTGGAT<br>GCTGTGAGGGAGGGAAGTGG	174
AGPAT6	FJ439669.1	CTGGGCATCTCCCTGACTGT<br>GATTCCATTGGTGTAGGGCTTG	198
DGAT1	AY116586.1	TGGACTACTCACGCATCAT<br>GTGGAAGAGCCAGTAGAAGAA	176
LPIN1	NM_001130734.1	CACATTTTGCCCACCCTT<br>GTGCCACGCTCGTTGACC	164
LPIN2	NM_001141987.1	CCTATGGAACTGGAACGA<br>TTGATGGAGTGGTAGAGCTTGG	141
PLIN2	NM_214200.2	CTCCTCAGTTCCAGCAAG<br>GGATAAAAGGGACCTACCAG	113
SREBP1	NM_214157.1	AGCGGACGGCTCACAATG<br>CGCAAGACGGCGGATTTA	121
INSIG1	NM_001244521.1	TGTCGTGGGCTTGCTCTA<br>GCACTGGCGTGGTTGATG	123
SCAP	AY705448.2	GCGGTGAGATTTTCCCCTAC<br>GCCAATGAGGATGATGCC	185
PPARα	DQ437887.1	CAGCGTGGCACTGAACATC<br>CTCCGATCACATTTGTCATAGAC	144
PPARγ	NM_214379.1	AGCCCTTTGGTGACTT<br>AGGACTCTGGGTGGTT	213

[1] Amplicon size in base pair (bp).
